# Health and Long Life

**Published:** 1862-05

**Authors:** 


**HEALTH AND LONG LIFE.**—Louis Cornaro, an Italian, was born about the year fourteen hundred and sixty-six, and died in fifteen hundred and sixty-five. He was born a feeble, weakly child. As he grew up he "addicted himself to all kinds of intemperate living," and at the age of forty was about to die, his physicians having abandoned all hope of his restoration. By some means he was induced to make an effort to restore his health by restraining and regulating his immoderate appetites. To this end, he promptly laid down for his observance a system of sober living, to which he steadily adhered for sixty years, with the result of regaining his health, his fortune, and his social position, that of a nobleman. On two different occasions he was induced, by the solicitations of friends, to increase the amount of his daily food and drink by a few ounces, but on both he was thrown into a serious illness, and no argument thereafter could ever induce him to transcend his habits.

This is a most instructive and encouraging narration to those who, at the age of forty years, find themselves in ill health. Naturally of a weak constitution, further impaired by long years of dissolute and drunken habits, poor and degraded, he seemed to have every thing to contend against; and yet, by resolute, persistent self-denial, he regained health, and wealth, and a high position in the councils of his country, eventually dying, without a sigh or pain, a hundred years old! It is instructive to know how these results were brought about, for doubtless they could be repeated an indefinite number of times by persons possessing the same elements of character, the most prominent of which were:

A heroic self-denial.

Indomitable industry.

A genial nature.

The last item is more properly a result of that vigorous health which uniformly attends a temperate, busy life. The good-hearted Italian denied himself the ordinary pleasures of eating and drinking. He drank nothing but the mild wine of the country, three quarters of a pint daily, (twelve ounces or twenty-four tablespoonfuls,) and ate three quarters of a pound of plain, solid food—such as bread, meats, vegetables, and fruits. He was active, energetic, and industrious. He went to work and became rich by agriculture. He possessed a generous nature. His heart was full of sympathy for others. Hence, when he became rich, he busied himself in improving the condition of those around him. In proof of which, he says in a letter to Cardinal C——, a relative, dated April 2d, 1542:

"I was weakly at my birth, and of a very feeble constitution, which I further injured by great irregularities. Being convinced of my errors, I commenced by reforming myself as to those most hurtful to me, and continued to shun disorderly courses until I acquired the perfect health which I now enjoy. I then regained the rank of noble in my native country, and by my own exertions have made myself rich. My wealth has been drawn from agriculture, a laudable occupation. At the same time I have incurred large expenses, but have never denied myself the enjoyments and recreations which are suitable to the rank of a noble. These expenditures were for building a church dedicated to God, and for the means of draining stagnant waters, and dissipating the unwholesome vapors and exhalations which existed around my villa, and rendered it impossible to rear children. Thus I have not only enriched myself, but have contributed to enrich numbers who were my agents and tenants. I have also used my means to promote the liberal arts, and have expended thousands upon thousands of crowns in constructing splendid edifices, and laying out beautiful gardens. Have I not a right to term myself happy when I am in the possession of the three blessings, health, nobility, and wealth, with the added consolation that the latter has been acquired by the most honorable of pursuits, and used with a becoming liberality, especially as I have a good son-in-law attached to the Court, and who has brought me three grandchildren, little angels in miniature?"

Another example of the power of temperance in eating was found in the history of a great name, of which there remain many loving recollections by some yet living in or near New-York City, who vividly remember the power of his eloquence, and what crowds flocked to hear him at the old Broadway Tabernacle, the building being filled to its utmost capacity, night after night, for weeks in succession. He had a herculean frame, and gave many a proof, before his embracing of Christianity, of personal courage and fearlessness. But he was an epileptic—that is, he knew the tendencies of his system were in that direction, and that the only method of preventing the attacks was the most rigid control of the appetite. His medical knowledge placed this truth so distinctly before his mind, and his self-control was so heroic, that, in speaking of it on one occasion to those very dear to him, he said: "I have been hungry for two years." This was exhibiting a rational self-denial worthy of all admiration, and is a lesson to that vast multitude which no man can number, who have not enough force of character, of moral courage, to prevent their yielding to their appetites three times a day; not merely to the extent of satiety, but of actual repletion; really eating, and that habitually, more than nature needs. Man is like a pig: when he is hungry, he gets quarrelsome, ill-natured, snappish; but in one respect he is unlike a pig—that animal, with all its love of filth, ceases to eat when it is no longer hungry. Reader, did you never eat to make it even?—take some more bread because you had a little butter on your plate? or take a little more sauce or gravy (called "essence" in the higher spheres) because a bit of bread was left, and you did not want to leave it? That was waste; it was more than waste, because it not only did you no good, but a positive injury. The great name mentioned in this article, under the influence of the gnawings of hunger, voluntarily endured, was one of the loveliest of men in his disposition we ever encountered, for we once lived under the same roof with him; yet to be hungry voluntarily for two years, and at the same time to have been so affectionate and so kind, that he has been known to give up his horse to a ragged, wayside traveler, who seemed less able to "foot it" than himself — this is the type of persistent and intelligent heroism which was manifested by the great author of the *Cause and Cure of Infidelity*, and it is a powerful argument as to the efficiency of a properly regulated diet in controlling diseases.

These histories are very suggestive. But let the reader bear in mind, that temperance in eating and drinking will not of themselves secure like results in any given case; this must be conjoined with such out-door activities as involve mental interest and exhilaration. In plainer language, temperance must be accompanied with some highly remunerative open-air employment, in which our benevolences are brought largely into requisition.

